# Investigation of the optimal b-value to detect breast tumors with diffusion weighted imaging by 1.5-T MRI

**DOI:** 10.1186/1470-7330-14-11

**Published:** 2014-04-22

**Authors:** Takayuki Tamura, Shigeru Murakami, Kumiko Naito, Tamaki Yamada, Takashi Fujimoto, Takeshi Kikkawa

**Affiliations:** 1 Department of Radiology, Hiroshima Atomic Bomb Casualty Council, Health Management & Promotion Center, 3-8-6 Sendamachi, Naka-ku, Hiroshima 730-0052, Japan; 2 Department of Surgery, Hiroshima Asa City Hospital, 2-1-1 Kabe-minami, Asakita-ku, Hiroshima 731-0293, Japan

## Abstract

**Background:**

Previous studies have reported that the signal attenuation of diffusion-weighted magnetic resonance imaging (DWI) for normal breast tissue and tumor were well fitted by a monoexponential and a biexponential function, respectively. The aim of this study was to investigate the optimal b-value to detect breast tumors from DWI signal attenuations.

**Methods:**

Sixty-four subjects with breast cancer underwent DWI using six b-values up to 3500 s/mm^2^. The signal attenuations of normal breast and tumor were fitted by mono- and biexponential functions, respectively. The maximum contrast b-values were estimated and compared in terms of frequency.

**Results:**

In almost all cases, the contrast increased with a b-value from 0 to approximately 1500 s/mm^2^. For b > 1500 s/mm^2^, the contrast decreased. The highest contrast b-value in the range of 0 to 2500 s/mm^2^ most frequently was b = 1500 and the next most frequent was 1400 s/mm^2^. Comparing sensitivity and specificity between b = 700 and b = 1400 s/mm^2^, b =1400 s/mm^2^ was slightly superior.

**Conclusion:**

Based on these results, DWI with a b-value of approximately 1400-1500 s/mm^2^ is recommended for optimizing breast tumor detectability.

## Background

MRI is the most sensitive imaging modality for the detection of breast cancer; however, the reported specificity is variable [1]. Generally, breast MRI diagnosis emphasizes tumor shape, margins, internal enhancement, and dynamic pattern using images acquired after a bolus injection of contrast media [2]. Furthermore, it had been reported that adding information from diffusion-weighted imaging (DWI) increases the specificity [3,4]. DWI can be used to distinguish between tissues by detecting differences in the diffusion properties of their water molecules. It is used to detect malignant tumors because the diffusion of water molecules in a malignant tumor is restricted and therefore the transit distances have shorter than in normal parenchyma [5-10]. DWI is often used in breast imaging, and many reports demonstrate its clinical utility [3,4,11-23]. In a meta-analysis of DWI of breast tumors performed at 1.5 T, Tsushima *et al. *[23] reported a sensitivity of 0.89 and specificity of 0.77, comparable to the results of the meta-analysis of contrast-enhanced breast MR imaging reported by Peters *et al. *[20] with a sensitivity of 0.90 and specificity of 0.72. Several studies have reported that the apparent diffusion coefficient (ADC) is useful in discriminating between malignant and benign breast tumors [3,11-23], but the threshold value of ADC for such discrimination can range from 1.1 to 1.6 × 10^−3^ mm^2^/s, and it is affected by the maximum b-value [23]. These ADCs were calculated by the following monoexponential equation using 2 or 3 b-values up to 1500 s/mm^2^,

(1) $$\mathrm{Sb}/S0=\exp\;\left(-\mathrm{bD}\right)$$

where *S0* and *Sb* are the signals without and with diffusion sensitizing gradients, respectively; *b* is the b-value, and *D* is the ADC [24]. However, the application of Equation (1) for measuring diffusion *in vivo* is problematic. The actual signal attenuation of breast tumors is non-monoexponential [25]; therefore, calculated ADCs using different b-values are also different [23].

Comparing the sensitivity and specificity of ADC using b = 0–1000 s/mm^2^ with a 250 s/mm^2^ interval to perform 1.5-T MR imaging, Pereira *et al. *[26] reported that the ADC calculated from b = 0 and 750 s/mm^2^ was slightly better than other b-value combinations for distinguishing malignant from benign. In a similar study using 3.0 T MRI, Bogner *et al. *[27] reported that the combination of b = 50 and 850 s/mm^2^ was slightly better, and using b-values over 1000 s/mm^2^ could lead to overdiagnosis and was not appropriate. However, Ochi *et al. *[3] reported that ADC values calculated from b = 1500 s/mm^2^ were useful to improve the diagnostic accuracy for malignant tumors and benign lesions, especially for noninvasive ductal carcinoma (NIDC) versus fibrocystic changes, except in cases of ductal hyperplasia. These differing results suggest that the optimum b-value for calculating the ADC has not yet been agreed upon. Additionally, it is well known that there is overlap between the ADCs of malignant and benign neoplasms [23], and although ADC reflects cell density, it does not directly reflect the character of the malignancy [11,19,28]. Proliferative tumors, both benign and malignant, exhibit high signal in DWI [22].

Another aim of DWI is the detection of tumors in general [18,22]. The optimum b-value for tumor detection has also not yet been agreed upon. There have been many recent studies of DWI using b-values > 1000 s/mm^2 ^[3,12,21,25-29]. Higher b-values are able to apply weightings to more restricted diffusion but also cause decreased signal-to-noise ratio (SNR). Thus, excessively higher b-values do not improved tumor detection. In our previous study, we analyzed the DWI signal attenuation of normal mammary gland and breast tumors using multiple b-values up to 3500 s/mm^2^ and demonstrated that normal breasts and malignant tumors were well fitted by a monoexponential and biexponential function, respectively [25]. The aim of the current study is to investigate the optimum b-value to detect breast tumors, surveying multiple b-value DWI signal attenuations.

## Methods

### Subjects

This study was approved by our center’s Institutional Review Board (Hiroshima Atomic Bomb Casualty IRB, Trial registry number, 5) and all patients gave their informed consent. The subjects included 62 females with a total of 64 breast cancers (mean age = 56.0 years, age range = 33–81 years, mean tumor size = 2.17 ± 1.48 cm) and 38 normal controls (mean age 55.4 years, age range = 35–78 years). All subjects underwent breast magnetic resonance imaging (MRI) including DWI with multiple b-values. All tumors were excised and the final diagnosis established on the basis of histopathological examination. According to the World Health Organization classification, the 64 breast tumors were comprised of noninvasive ductal carcinoma (NIDC; n = 9), invasive carcinoma (IDC; n = 49), tubulo-lobular carcinoma (n = 3), mucinous carcinoma (n = 2), and medullary carcinoma (n = 1).

### MR imaging

**Figure 1:**
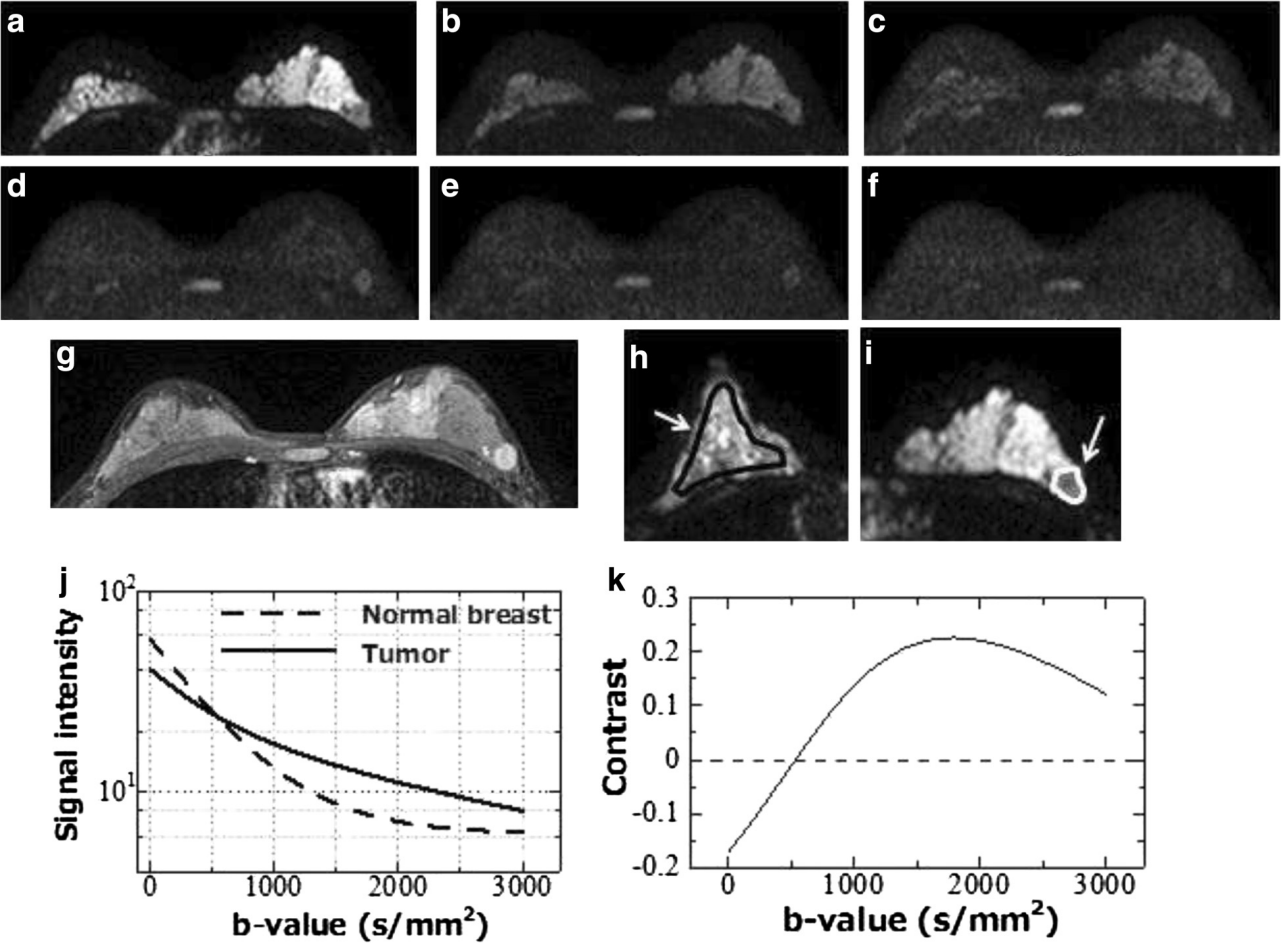
**DWIs of a 39-year-old woman with invasive ductal carcinoma of the left breast.** b-value = 0 **(a)**; 700 **(b)**; 1400 **(c)**; 2100 **(d)**; 2800 **(e)**; and 3500 **(f)** s/mm^2^ and 3D T_1_-weighted fast gradient-echo image at 70 second after contrast administration **(g)**. Illustrated positions of the ROIs of normal breast **(h)** and tumor **(i)** on the DWI. DWI signal attenuations of the normal breast and tumor **(j)** and derived contrast with function of b-value **(k)**.

All MR imaging was performed using a 1.5 T superconducting magnet (Gyroscan Achieva R.2.6; Philips Medical Systems, Best, The Netherlands) with a 7-channel SENSE breast coil. In the prone position, patients underwent diffusion-weighted axial imaging using the spin-echo type single-shot echo planar imaging (EPI) technique. We used the tetrahedral diffusion gradient technique [30] to suppress prolongation of the echo time (TE) and obtained four image series, from which the isotropic diffusion gradient strength images were generated. The six diffusion b-values ranged from 0 to a maximum of 3500 s/mm^2^ with an interval of 700 s/mm^2^ (Figure 1a–f). The diffusion gradient duration (*δ*) was 37 ms with an interval (*Δ*) of 48.5 ms; both were fixed at every b-value, with only the diffusion-sensitizing gradient strength (*G*) changed with each change in the b-value. Thus, the diffusion duration (*Δ* - *δ*/3) was constant at 36 ms. The other parameters used for the DWI sequence are shown in Table 1. After the DWI, bilateral axial examinations consisting of a fast spin-echo T_2_-weighted sequence with fat suppression and a T_1_-weighted gradient-echo sequence were performed. For the dynamic contrast-enhanced examination, 3D T_1_-weighted fast gradient-echo sequences were acquired before, 70 seconds (early phase) (Figure 1g), and 5 minutes (delayed phase) after the bolus injection of gadolinium contrast media (dose: 0.2 ml/kg, rate: 2 ml/s). During the dynamic examination (3 minutes after the injection), an additional high-resolution 3D T_1_-weighted fast gradient-echo sequence was acquired (Table 1).

**Table 1:** Scan parameters of breast MRI study

	**Technique**	**TR/TE/FA**	**Matrix**	**FOV**	**Thickness/Gap**	**NEX**	**SENSE**	**Fat Sat.**	**Scan time**
DWI	2D-SE-EPI	5600/99/90	128 × 114(256R)	280 mm	4/0.4 mm	3	Factor 2.0	SPAIR	4 m 40 s
T2WI	2D-FSE	6200/80/90	256 × 198(256R)	280 mm	4/0.4 mm	2	Factor 2.0	SPAR	1 m 58 s
T1WI	2D-GRE	268/5.5/80	256 × 198(256R)	280 mm	4/0.4 mm	1	Factor2.0	Non	1 m 27 s
Dynamic	3D-Fast-GRE	7.7/3.8/12	352 × 334(512R)	280 mm	1.2/-0.6 mm	1	Factor2.0	SPAIR	70 s × 3
High-Resolution	3D-Fast-GRE	8.6/4.3/12	512 × 496(512R)	280 mm	1.21-0.6 mm	1	Factor2.0	SPAIR	3 mls

Prior to the clinical study, we determined DWI signal attenuation of 4% CuSo_4_-doped saline to confirm the linearity of the diffusion gradient with each b-values. The scan parameters were identical to those of the clinical study without b-values. The number of diffusion b-values was changed to sixteen from six with an interval of 233 s/mm^2^, thereby, scan time was extended to 35 min 20 s.

### Regions of interest

We displayed and analyzed DWI images using “ImageJ” (National Institutes of Health, Bethesda, Maryland, USA; http://rsbweb.nih.gov/ij/), a freely available DICOM viewer. We placed a region of interest (ROI) in a target tumor and in the contralateral normal breast on DWI. In tumors, we confirmed the orientation using the DWI of b = 700 and 1400 by comparing early phase images of the dynamic study. ROIs were smaller than the mass size and excluded areas of normal-appearing tissue (mean size = 112 ± 96.7 pixels). In normal breasts, ROIs were made in the nipple level’s mammary grand as large as possible excluding the area of fat (mean size = 503.4 ± 417.2 pixels) (Figure 1h). We plotted the DWI signal using in-house software developed in a commercial analysis package (Matlab v.7.8, MathWorks Inc., Natick, MA, USA) and fitted the data using the Levenberg-Marquardt algorithm [31] for malignant tumors as follows:

(2) $$\mathrm{Sb}/S0=f_{\mathrm{fast}}\exp\;\left(-bD_{\mathrm{fast}}\right)\;+\;\left(1-f_{\mathrm{fast}}\right)\;\exp\;\left(-bD_{\mathrm{slow}}\right)\;+\mathrm{BG}$$

where *f* is the fraction of each component, the subscripts indicates the fast and slow components and *BG* is background [32], and used equation (1) for normal mammary glands.

### Tumor and normal breast contrast

Using the parameters calculated from signal fitting, we reproduced the signal decay of tumor and normal breast tissue and calculated the contrast between them from b = 0 to 3000 s/mm^2^ with an interval of 100 s/mm^2^ from the following equation:

(3) $$\begin{array}{l} \mathrm{Contrast}=\left\{\mathrm{Signal}\left(\mathrm{tumor}\right)-\mathrm{Signal}\left(\mathrm{normal}\;\mathrm{breast}\right)\right\}/\\ \;\left\{\mathrm{Signal}\left(\mathrm{tumor}\right)+\mathrm{Signal}\left(\mathrm{normal}\;\mathrm{breast}\right)\right\} \end{array}$$

In each case, we investigated the maximum contrast b-value and counted the frequency of maximum contrast b-values in all cases.

### Sensitivity and specificity of DWI

We evaluated the sensitivity and specificity using b = 700 and 1400 s/mm^2^ of DWI by three observers (TT, MS, and NK with 11, 10, and 9 years of expertise in breast MRI diagnosis, respectively). The subjects were a total of 100 cases including the 62 patients (64 breast cancers) and the additional 38 normal cases. A total of 200 images (400 breasts) were randomly sorted and evaluated the ability of tumor detection and localization. The results were compared using the chi-square test (95% confidence interval); a *p* value < 0.05 was considered significant.

## Results

### Tumor and normal breast contrast

**Figure 2:**
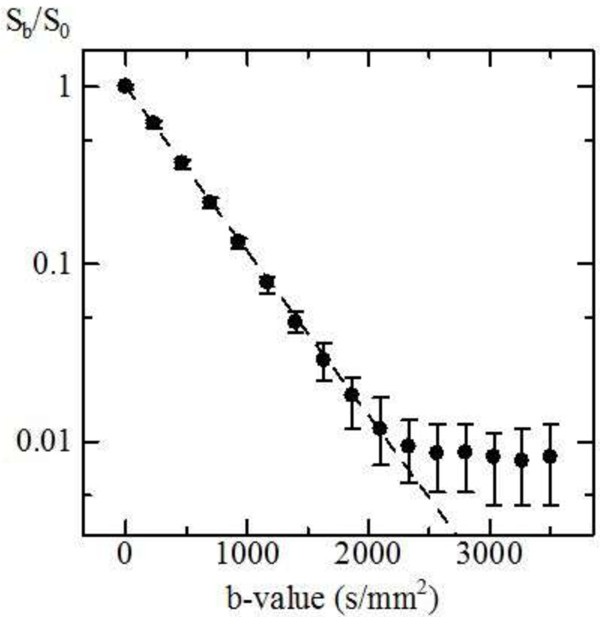
**Diffusion attenuation in 4% CuSo**_**4**_**-doped saline at 22°C indicating a linear signal decay until it reached background level.**

The signal decay as a function of b factor for the 4% CuSo_4_-doped saline (22°C), shown in Figure 2, was found to be monoexponential, as expected. The diffusion coefficient obtained from the fit was 2.10 × 10^−3^ mm^2^/s, in close agreement with the value obtained by Niendorf *et al. *[33] of 2.04 × 10^−3^ mm^2^/s.

**Figure 3:**
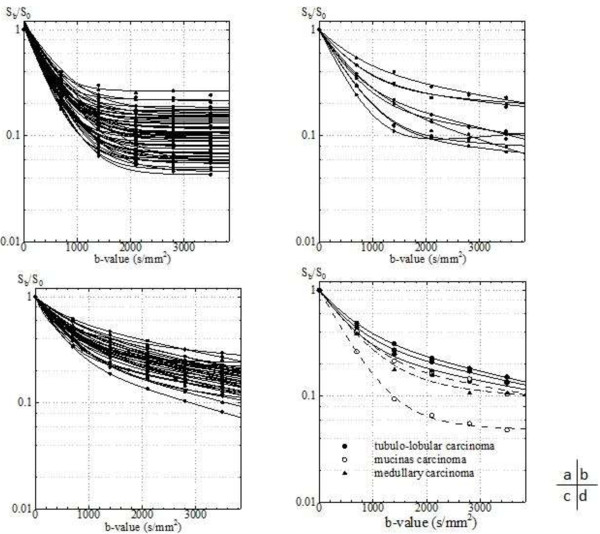
Signal attenuation of all cases of normal breasts (a), NIDC (b), IDC (c) and special type tumors (d).

All of the normal mammary gland and tumor DWI signal attenuations are shown in Figure 3. All normal breast signal decays were fitted by the monoexponential equation (1) and tumors were fitted by the biexponential equation (2). The mean ADC of normal breast tissue was 2.48 ± 0.33 × 10^−3^ mm^2^/s. In malignant tumor, derived parameters from the biexponential fitting are displayed in Table 2.

**Table 2:** Derived parameters from the biexponential fitting in malignant tumors

	**n**	***f***_***fast***_	***D***_***fast***_	***D***_***slow***_
NIDC	9	0.717 ± 0.114	2.07 ± 0.319	0.179 ± 0.10
IDC	49	0.624 ± 0.125	2.08 ± 0.48	0.192 ± 0.09
Special type	6	0.673 ± 0.172	2.05 ± 0.32	0.203 ± 0.10

*f*_*fast*_: fast component fraction, *D*_*fast*_: fast component ADC (×10^−3^ mm^2^/s), *D*_*slow*_: slow component ADC (×10^−3^ mm^2^/s), NIDC: Non-invasive ductal carcinoma, IDC: Invasive ductal carcinoma.

**Figure 4:**
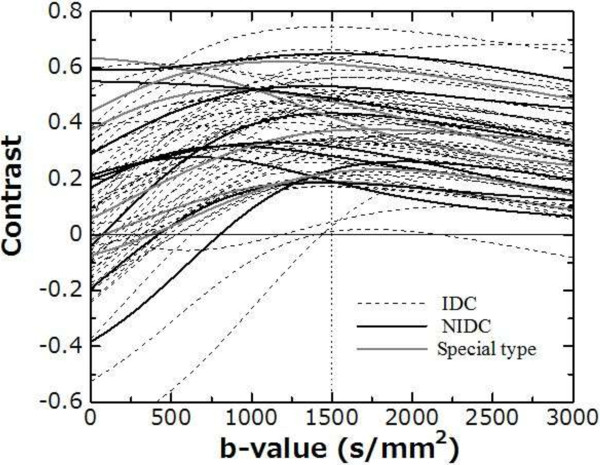
Contrast curves between tumor and normal breast for all cases.

**Figure 5:**
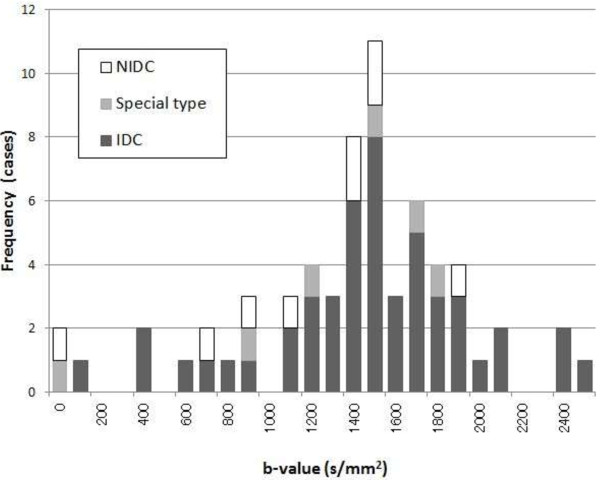
Frequency of maximum contrast b-value between tumor and normal breast.

Figure 4 shows the normal breast-to-malignant tumor contrast for all case. In almost every case, the contrast increased with the b-value from b = 0 to around 1500 s/mm^2^; for b > 1500 s/mm^2^, the contrast decreased. Figure 5 shows the frequency of the highest contrast b-values. Almost every case had the highest contrast in the range of b = 0–2500 s/mm^2^ and the most frequent were b = 1400–1500 s/mm^2^.

### Sensitivity and specificity of DWI

Table 3 gives the sensitivity and specificity of DWI performed with b = 700 and 1400 s/mm^2^. There is no difference between b = 700 and 1400 s/mm^2^ in terms of sensitivity and specificity for observer A; in the other two observers, both the sensitivity and specificity of b = 1400 s/mm^2^ were slightly better than for b = 700 s/mm^2^. Consequently, the mean sensitivity of b = 700 and 1400 s/mm^2^ was 0.871 and 0.892, respectively, and the mean specificity was 0.863 and 0.912, respectively. For both criteria, b = 1400 s/mm^2^ was slightly better than b = 700 s/mm^2^. However, there were no significant differences in either sensitivity or specificity between b = 700 and b = 1400 (*p* = 0.504 for sensitivity, *p* = 0.197 for specificity).

**Table 3:** **The sensitivity and specificity of DWI using b = 700 and 1400 s/mm**^**2**^

	**Sensitivity**		**Specificity**	
**Observer**	**b = 700**	**b = 1400**	**b = 700**	**b = 1400**
A	0.871	0.871	0.853	0.853
B	0.871	0.903	0.794	0.912
C	0.871	0.903	0.941	0.971
Mean	0.871	0.892	0.863	0.912

## Discussion

In this study, we assessed the DWI signal attenuation of normal breasts and tumors up to b =3500 s/mm^2^ using multiple b-value DWI data, and calculated the maximum contrast between normal breast and tumor. The frequencies of the maximum contrast b-values between malignant tumor and normal breast were widely distributed within the range of b = 0 to b = 2500 s/mm^2^; the most frequent b-value was b = 1500 s/mm^2^ (16.9%, 11/65) and the next was b = 1400 s/mm^2^ (12.3%, 8/65) (Figure 5).

The tumor-to-normal breast contrast of almost all cases increased with a b-value from b = 0 to approximately 1500 s/mm^2^, and decreased with b > 1500 s/mm^2^ (Figure 4). Increasing the b-value caused the DWI signal to decrease and in almost all cases of normal breast, the signal declined until it reached the background noise level at around b = 1000–1500 s/mm^2^. In addition, for b ≥ 1500 s/mm^2^, normal breast signals were constant as the background and tumor signals were residual (Figure 3). Consequently, the maximum contrast b-values were of high frequency at approximately b = 1500 s/mm^2^.

**Figure 6:**
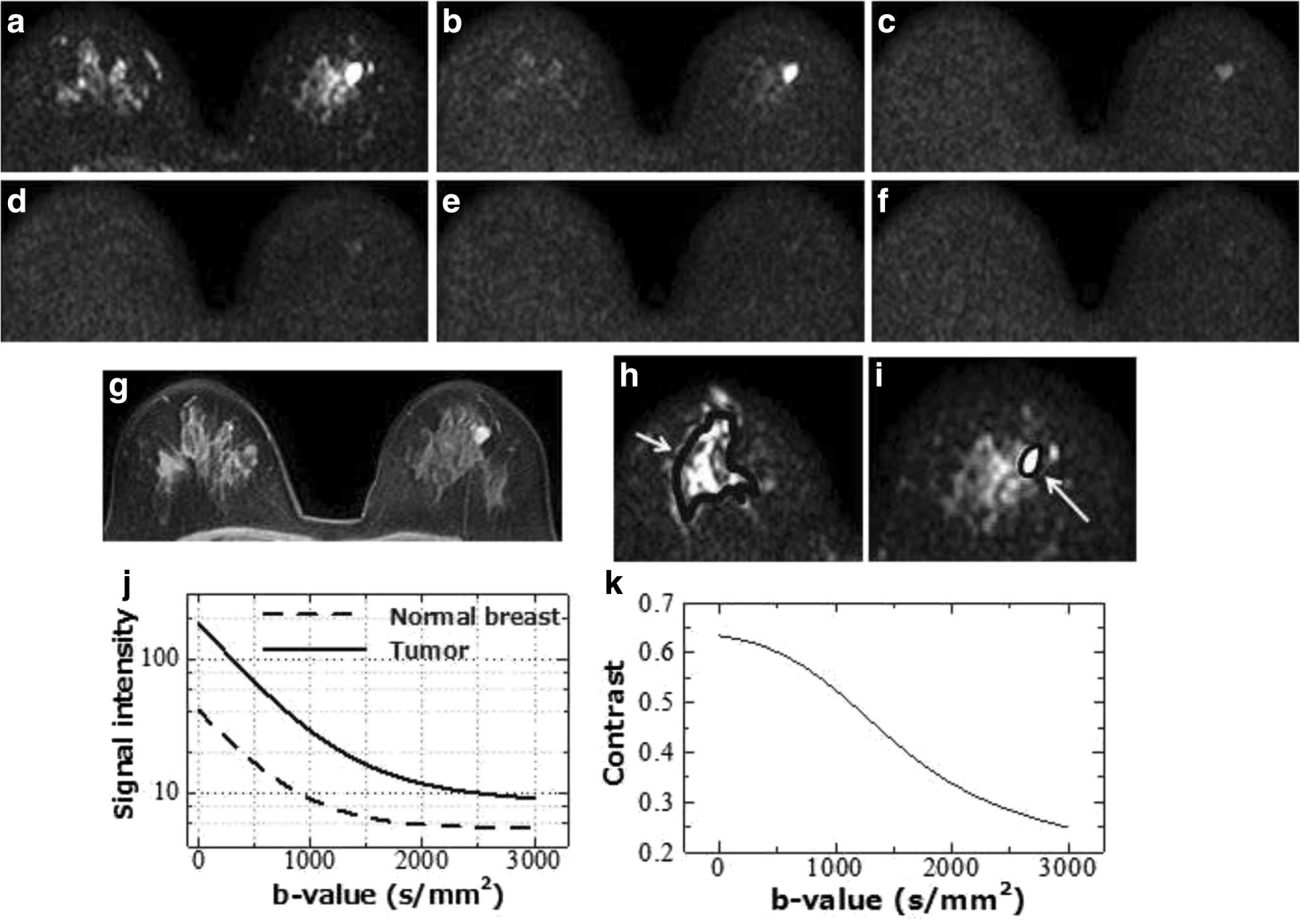
**DWIs of a 59-year-old woman with mucinous carcinoma of the left breast.** b-value = 0 **(a)**; 700 **(b)**; 1400 **(c)**; 2100 **(d)**; 2800 **(e)**; and 3500 **(f)** s/mm^2^ and 3D T_1_-weighted fast gradient-echo image at 70 second after contrast administration **(g)**. Illustrated positions of ROIs of normal breast **(h)** and tumor **(i)** on the DWI. DWI signal attenuations of the normal breast and tumor **(j)** and derived contrast with function of b-value **(k)**.

**Figure 7:**
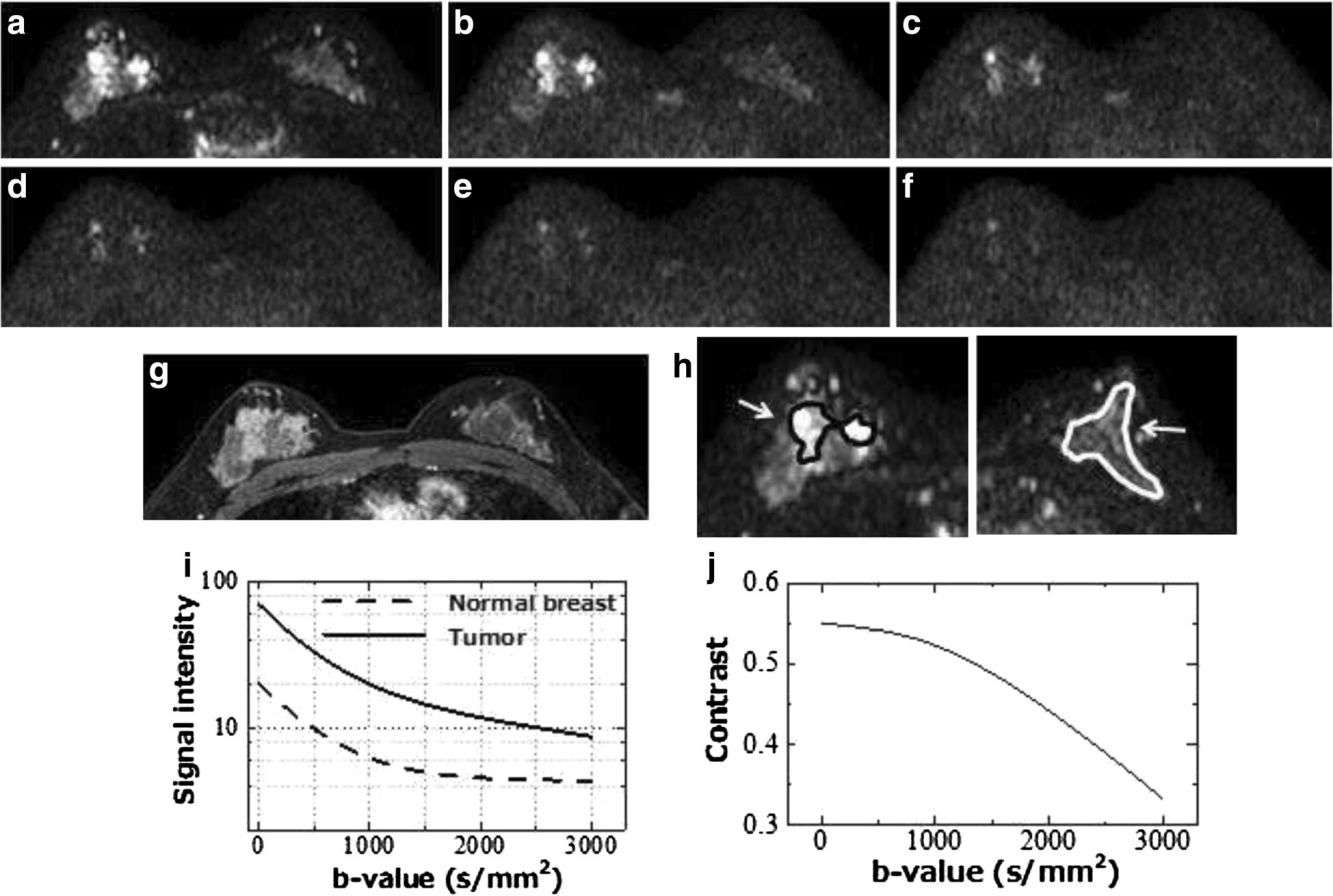
**DWIs of a 39-year-old woman with NIDC of the right breast.** b-value = 0 **(a)**; 700 **(b)**; 1400 **(c)**; 2100 **(d)**; 2800 **(e)**; and 3500 **(f)** s/mm^2^ and 3D T_1_-weighted fast gradient-echo image at 70 second after contrast administration **(g)**. Illustrated positions of ROIs of tumor **(h)** and normal breast **(i)** on the DWI. DWI signal attenuations of the normal breast and tumor **(i)** and derived contrast with function of b-value **(j)**.

There were two cases in which the highest contrast b-value was estimated to be 0 s/mm^2^. These two cases were a NIDC and a mucinous carcinoma and both were of very high intensity in the T_2_-weighed images and also displayed high intensity in DWI b = 700 and b = 1400 s/mm^2^ (Figures 6 and 7).

Subsequently, we examined the sensitivity and specificity of DWI at b = 700 and 1400 s/mm^2^, which was where the maximum contrast was seen in only two cases and where the higher frequency was observed, respectively. This was done to confirm whether the tumor detectability could be evaluated or not by using the optimal b-value (Table 3). As a result, the mean sensitivity and specificity of the three observers of the b = 1400 s/mm^2^ data were slightly superior to those for b = 700 s/mm^2^. By comparing the sensitivity and specificity, unfortunately, there was no statistically significant difference (*p* = 0.504 for sensitivity, *p* = 0.197 for specificity). However, for DWI, the residual signal of normal breast tissue suppresses the conspicuity of tumor. The DWI at b = 1400 s/mm^2^, without residual signal from normal tissue, is more likely to detect tumor.

In a similar study using 6 b-values up to b = 3000 s/mm^2^ and a 3.0 T MRI, Takanaga *et al. *[29] reported that the maximum contrast b-value between tumor and normal breast tissue was 1500 s/mm^2^ and they concluded that the decline of b-values for normal-breast-to-background level is higher than 1.5 T, because the SNR of 3.0 T MRI is higher than that of 1.5 T. As a result, the highest contrast b-value for 3.0 T might be higher than that of 1.5 T. However, the b-value using 1.5 T MRI was the same in our study. Matsuoka *et al*. [34] reported that there was no significant difference for breast tumor ADCs between 1.5 T and 3.0 T. We consider that the DWI signal attenuation does not depend on the magnetic field strength; consequently, there was no difference between the maximum contrast b-values for 1.5 and 3.0 T.

There are some limitations in the present study. First, we reproduced the DWI signal decay using only 6 b-value images because of limitations in examination time, and less data may cause incorrect signal decay reproduction. As the decay of normal breast declines to the background noise level until the lower b-value, there were only two or three data points generated during decay. Thus, there is a possibility that accurate signal attenuation cannot be reproduced. Furthermore, the shape of decay is affected by the ROI’s position and shape. Because of the heterogeneity within the tumor, DWI signal attenuation is different depending on the ROI’s position and shape. In this study, we set the ROIs to include the entire area of the tumor, thus the contrast between tumor and normal tissue might be underestimated and the estimated maximum contrast b-value may differ from actual values. Even with the presence of the abovementioned uncertainties, we speculate that maximum contrast b-values exist around b = 1500 s/mm^2^ with high frequency.

As a result of this study, the optimum b-value to detect breast tumors depends on the case, and so we consider it to be impossible to determine a single optimal b-value. Generally, the b-value is selected to be less than 1000 s/mm^2^ in breast DWI, although we recommend the use of approximately b-1500 s/mm^2^ to improve the diagnostic performance. However, we do not recommend the use of high b-values greater than 1000 s/mm^2^ only, because there are some reports that the accuracy of ADC to distinguish malignant from benign tissue is greatest when using values of b < 1000 s/mm^2 ^[26,27]. Consequently, we recommend using a combination of b = 750–850 s/mm^2^ for calculating ADC and b = 1400–1500 s/mm^2^ for detecting tumors. Using multiple b-value DWI causes prolongation of the scan time and so it may not be suitable to perform these steps in the limited time available. Additionally, it is impossible to set multiple b-values with some vendors’ machines, and selecting different b-values can cause more change in the TE. Higher b-values cause decreased SNR because of diphase due to water molecule diffusion; prolongation of TE will be further conducive to this. Therefore, tumor detection might become difficult under such conditions. Thus, we hope that advancement of technology for employing multiple b-values will enable their use in all vendors’ machines in a shorter TE with higher SNR.

## Conclusion

We evaluated the malignant tumor/normal mammary gland contrast of 1.5 T breast DWI using six b-values up to 3500 s/mm^2^ and investigated the optimal b-value to detect breast cancer. As a result, the maximum contrast b-value was distributed around b = 0 to 2500 s/mm^2^, and b = 1400 and 1500 s/mm^2^ were the most frequent. Comparing sensitivity and specificity between b = 700 and 1400 s/mm^2^, b = 1400 s/mm^2^ was slightly superior to b = 700 s/mm^2^. From these results, DWI with a b-value of 1400–1500 s/mm^2^ is recommended for improving breast tumor detectability. Given that there are some reports that b = 750–850 is suitable for distinguishing malignant from benign tissue using ADC, we recommend performing multiple b-value DWI combinations (b = 750–850 s/mm^2^ and 1400–1500 s/mm^2^) for breast DWI.

## Competing interest

The authors declare that they have no competing interests.

## Authors’ contributions

TT carried out the Analysis and interpretation of data and drafted the manuscript. MS and NK participated in the design of the study and performed the image evaluation. FT, YT and KT participated data correction and helped to draft the manuscript. All authors read and approved the final manuscript.
